# Correction to *Lancet Psychiatry* 2018; 5: 987–1012

**DOI:** 10.1016/S2215-0366(18)30488-7

**Published:** 2019-01

*GBD 2016 Alcohol and Drug Use Collaborators. The global burden of disease attributable to alcohol and drug use in 195 countries and territories, 1990–2016: a systematic analysis for the Global Burden of Disease Study 2016.* Lancet Psychiatry *2018;* 5: *987–1012*—The Creative Commons Open Access license has changed to CC BY 4.0. This correction has been made to the online version as of Dec 19, 2018.

